# Bladder cancer stage-associated hub genes revealed by WGCNA co-expression network analysis

**DOI:** 10.1186/s41065-019-0083-y

**Published:** 2019-01-28

**Authors:** Yu Di, Dongshan Chen, Wei Yu, Lei Yan

**Affiliations:** 1 Department of Urinary Surgery, Qilu Hospital, Jinan, Shandong province China; 2 Department laboratory of cardiovascular center of Shandong province, Jinan, Shandong province China; 3 Lanzhou medical college of Lanzhou University, Lanzhou, Gansu province China

**Keywords:** Bladder cancer, Weighted gene co-expression network analysis (WGCNA), Tumor staging, Hub gene identification

## Abstract

**Background:**

Bladder cancer was a malignant disease in patients, our research aimed at discovering the possible biomarkers for the diseases.

**Results:**

The gene chip GSE31684, including 93samples, was downloaded from the GEO datasets and co-expression network was constructed by the data. Molecular complex detection(MCODE) was used to identify hub genes. The most significant cluster including 16 genes: *CDH11*, *COL3A1*, *COL6A3*, *COL5A1*, *AEBP1*, *COL1A2*, *NTM*, *COL11A1*, *THBS2*, *COL8A1*, *COL1A1*, *BGN*, *MMP2*, *PXDN*, *THY1*, and *TGFB1I1* was identified. After annotated by BiNGO, they were suggested associated with collagen fibril organization and blood vessel development. In addition, the Kaplan Meier curves were obtained by UALCAN. The high expression of *THY1*, *AEBP1*, *CDH11*, *COL1A1*, *COL1A2*, *COL11A1*, *MMP2*, *PXDN*, *BGN*, *COL5A1, COL8A1*, and *TGFB1I1* indicated poor prognosis of the patients(*P* < 0.05). Finally, we examined genes’ expression between low and high tumor stage by the Wilcoxon test(P < 0.05), *TGFB1I1* was excluded.

**Conclusion:**

*THY1, AEBP1, CDH11, COL1A1, COL1A2, COL11A1, MMP2, PXDN, BGN, COL5A1, COL8A1* associated with the tumor stage as well as tumor patients’ prognosis*. COL5A1, COL8A1*(*P* < 0.01) may serve as therapeutic targets for the disease.

## Backgrounds

Bladder cancer was ranked fourth of the most common cancers in male, with an estimated 17, 240 deaths and 81, 190 new cases in 2018 cancer statistics [1]. The survival rate of bladder carcinoma sharply declined with the spreading of the tumor. The most common type of bladder cancer was transitional cell carcinoma, also called urothelial carcinoma. It was often diagnosed at an early stage, producing negative impacts on the patients daily life and wasting extensive social wealthy [2]. Currently, there were four types of standard treatment for bladder cancer including surgery, radiation therapy, chemotherapy, and immunotherapy; surgery was the primary method in clinical trials [3]. Transurethral resection (TUR) with fulguration was suitable for treating superficial bladder cancer while radical cystectomy was often rendered for patients with bladder cancer at the advanced stage in the situation of which the organs near the bladder such as the prostate and the seminal vesicles of a male patient and the uterus, the ovaries, and part of the vagina of a female need to be removed [4]. Partial cystectomy can be offered to patients who have a low-grade tumor that has invaded the bladder wall but was limited to one area of the bladder [5–7]. Biological therapy was an emerging revolution in the medical field, which involved the use of living organisms, substances derived from living organisms, or laboratory-produced versions of such substances to treat diseases. It can be used to treat cancer itself or the side effects of other cancer treatments [8]. Targeted cancer therapy was a type of biological therapy, which employs targeted-cancer-therapy drugs such as therapeutic antibodies to destroy cancer cells by interfering with a specific molecular target needed for cancer cell growth [9]. As such, it was important to find the critical genes which may be related to cancer growth and proliferation and thus can further affect the prognosis of patients. Weighted gene co-expression network analysis (WGCNA) was a systems biology method for describing the correlation patterns among genes across microarray samples, and can be used for finding modules of highly correlated genes, for summarizing such clusters using the module eigengene or an intramodular hub gene, for relating modules to one another and to external sample traits, and then the correlation networks can be used to identify candidate biomarkers or therapeutic targets [10]. It can be used to discover the genes for unknown function with biological processes, or for candidate disease or transcriptional regulatory work. Although it didn’t provide causality, the co-expression network can be used to identify regulatory genes underlying various phenotypes [11]. Multiple testing problems inherent in the microarray data analysis were resolved in WGCNA. Network approaches provided means to bridge the gap from individual genes to systems oncology [12]. It was not only suitable for mRNAs, but also for microRNAs and lncRNAs, for example, Giulietti, M et al. [13] have reported the expression of *LINC00675* and *LINC01133* lncRNAs associated with pancreatic cancer’s development and progression by co-expression network analysis. Zhou, X. G et al. [14] have found that hsa-miR-125b-5p, hsa-miR-145-5p, hsa-let-7c-5p, hsa-miR-218-5p, and hsa-miR-125b-2-3p were hub miRNAs related to prognosis as well as the pathological stage in colon cancer by the method. In fact, if the data met the requirements, WGCNA can be applied and certain information can be obtained. In our study, the WGCNA algorithm was employed to construct co-expression network, which was integrated with genetic information to explain the biological significance of the module genes, and then identified the hub genes of bladder cancer. Although it has been applied bladder cancer in several articles such as Li, S [15], Deng, S. P. [16], and Gaballah [17], and concluded by Giulietti, M [18], however, they choose to perform WGCNA only on DEG genes which may easily obtain the positive results, reduce the workload of calculation, and at the same time increase the possibility of leaving out the lowly expressed but highly correlated genes. In our research, all the possible genes were analyzed and our results would be more complete and reliable.

## Materials and methods

### Data collection

The gene chip GSE31684 [19] of bladder cancer with its clinical manifestation data was downloaded from the GEO database (https://www.ncbi.nlm.nih.gov/geo/). The platform was GPL570(Affymetrix Human Genome U133 Plus 2.0), and there were 93 bladder cancers samples with several clinical variables. A series of clinical traits are shown in Table 1. The raw data had been processed, and the gene expression matrix provided by the website was directly used for the analysis. More than 50,000 genes were entered into the BRB-arraytools (https://linus.nci.nih.gov/BRB-ArrayTools/) [20] for filtering: genes were excluded when less than 20% of expression data had at least a 1.6 -fold change in either direction from gene’s median value, and when the percent of data missing or filtered out exceeded 50%. The fold change value is not obtained by comparing the normal and tumor samples. It is a minimum fold-change filter, a parameter in BrB-arraytools, whose detail description can be found in the user manual in the website (https://linus.nci.nih.gov/BRB-ArrayTools/Documentation.html). Finally, a total of 13,222 genes passed the criteria. Compared to analyze the DEGs, our work requires a high equipment computer and carefully distinguish the false positive results, as mentioned above, its merit is obvious: the results would be more complete.

**Table 1:** The summary clinical information of the samples

Items	Counts value
Gender	
Male	68
Female	25
Age at diagnosis	69.13±
	10.15(years)
Survival Months	47.47±
	44.52(months)
Stage	
*pTa*	5
*pT1*	10
*pT2*	17
*pT3*	42
*pT4*	19
PLND Result	
*Negative*	49
*Positive*	28
*None*	16
Grade	
*High*	87
*Low*	6
Distant Lymphonodus Metastasis	
*none*	59
*yes*	34
Local Recurrence After Surgery	
0	73
1	20
Metastasis	
0	57
1	36
Smoking	
*Former*	56
*Never*	18
*Current*	19

### WGCNA analysis of the filtered genes

**Fig. 1:**
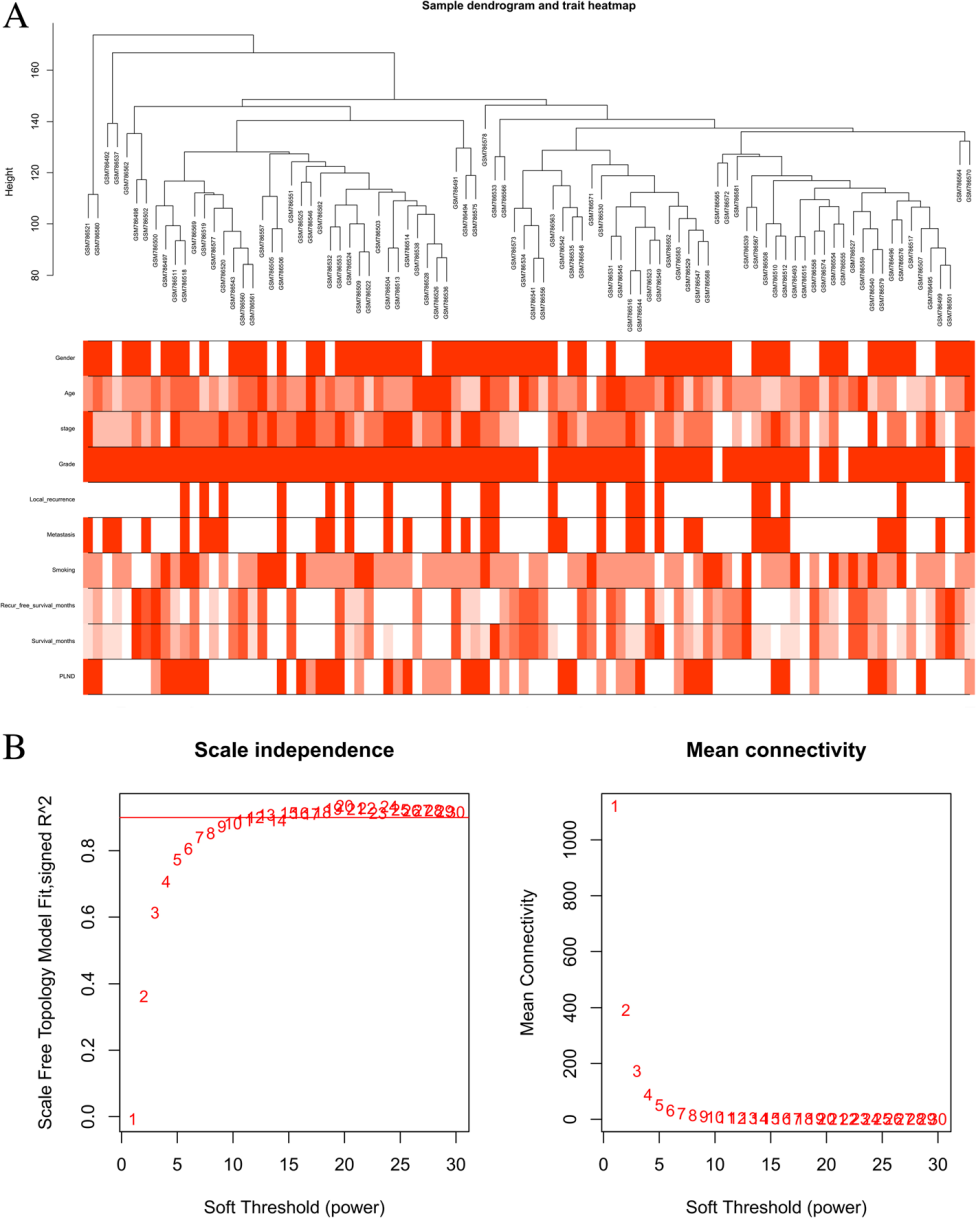
Sample dendrogram and soft-thresholding values estimation. **a** Sample dendrogram and trait heatmap. The cut height was set as 150 with four deviated samples of GSM786521, GSM786580, GSM786492 and GSM786537. The ten traits are respectively gender, age, stage, grade, local recurrence, metastasis, smoking, recurrence-free survival months, survival months and pelvic lymph node dissection (PLND, positive means local metastasis) (**b**) Scale independence and mean connectivity of various soft-thresholding values (β)

The “WGCNA” R package was used to construct a co-expression network for the filtered genes [21]. After employing the “hclust” function to the expression matrix evaluated by the average method, gene chips including GSM786521, GSM786580, GSM786492, and GSM786537 whose cluster height surpass 150 were identified deviated and thus excluded from further analysis (As shown in Fig. 1a). The other 89 samples were used to calculate the Pearson’s Correlation Matrices. The matrix of weighted adjacency was created by formula *a*_*mn*_ *= |c*_*mn*_*|*^*β*^ (*a*_*mn*_: adjacency between gene m and gene n, *c*_*mn*_: Pearson’s correlation, *β*:soft-power threshold). Afterward, the clinical trait data were loaded and the scale independence and mean connectivity were estimated. Additionally, the topological overlap measure(TOM) matrix, transformed by the adjacency matrix, was used to estimate its connectivity property in the network [22]. A hierarchical clustering dendrogram of the TOM matrix was constructed by the average distance with a minimum size threshold of 30 to classify the similar genes expression profiles into different gene modules. The different module eigengenes (MEs) and the clinical traits were then correlated**.** The gene significance (GS) quantifying associations of individual genes with the clinically interesting trait and the module membership (MM) which acted as the correlation between the module eigengenes and the gene expression profiles were calculated. As proved by previous research, if the GS and MM were highly correlated, the most important (central) elements in the modules were also tightly associated with the trait [23]. As such, they can be used to construct the network and identify the hub genes.

### Co-expression network construction and hub genes identification

The Cytoscape v3.6.0 was used to visualize the co-expression network in the module of interest [24]. At the same time, the network was analyzed by Molecular Complex Detection(MCODE) [25], a Cytoscape plugin that detected densely connected regions in networks that may represent molecular complexes. Algorithms for finding clusters, or locally dense regions, of a graph, were an ongoing research topic in computer science and were often based on network flow/minimum cut theory. The internal mechanism demonstrated that MCODE was suitable for co-expression network, where the proteins in the same complex often displayed high correlations and may be detected by the plugin. The parameters were set as follows. Degree cutoff: 2, node score cutoff: 0.2, cut style: haircut, k-core: 2, and max. Depth: 100. At the same time, WebGestalt was applied to find the transcriptional factors (TFs).

### Function annotation of the module of interest and the hub genes

The gene ontology (GO) analysis results of the genes in the module of interest were also provided by WebGestalt [26]. As a biological network gene ontology tool for functional enrichment analysis in various biological contexts [27], Bingo was used to analyze the hub genes with the following criteria: significant level:0.05; statistic test: binomial; multiple testing corrections: Benjamini and Hochberg False Discovery Rate correction.

### Survival analysis by TCGA data and results validation

UALCAN was a web portal for analyzing cancer transcriptome data [28]. It provided easy access to publicly available cancer transcriptome data and allowed users to identify biomarkers or to perform in silico validation of potential genes of interest. The overall survival of bladder cancer was conducted by UALCAN, and the expression levels of the hub gene in different stages were examined.

## Results

### WCGNA analysis and modules significance calculation

**Fig. 2:**
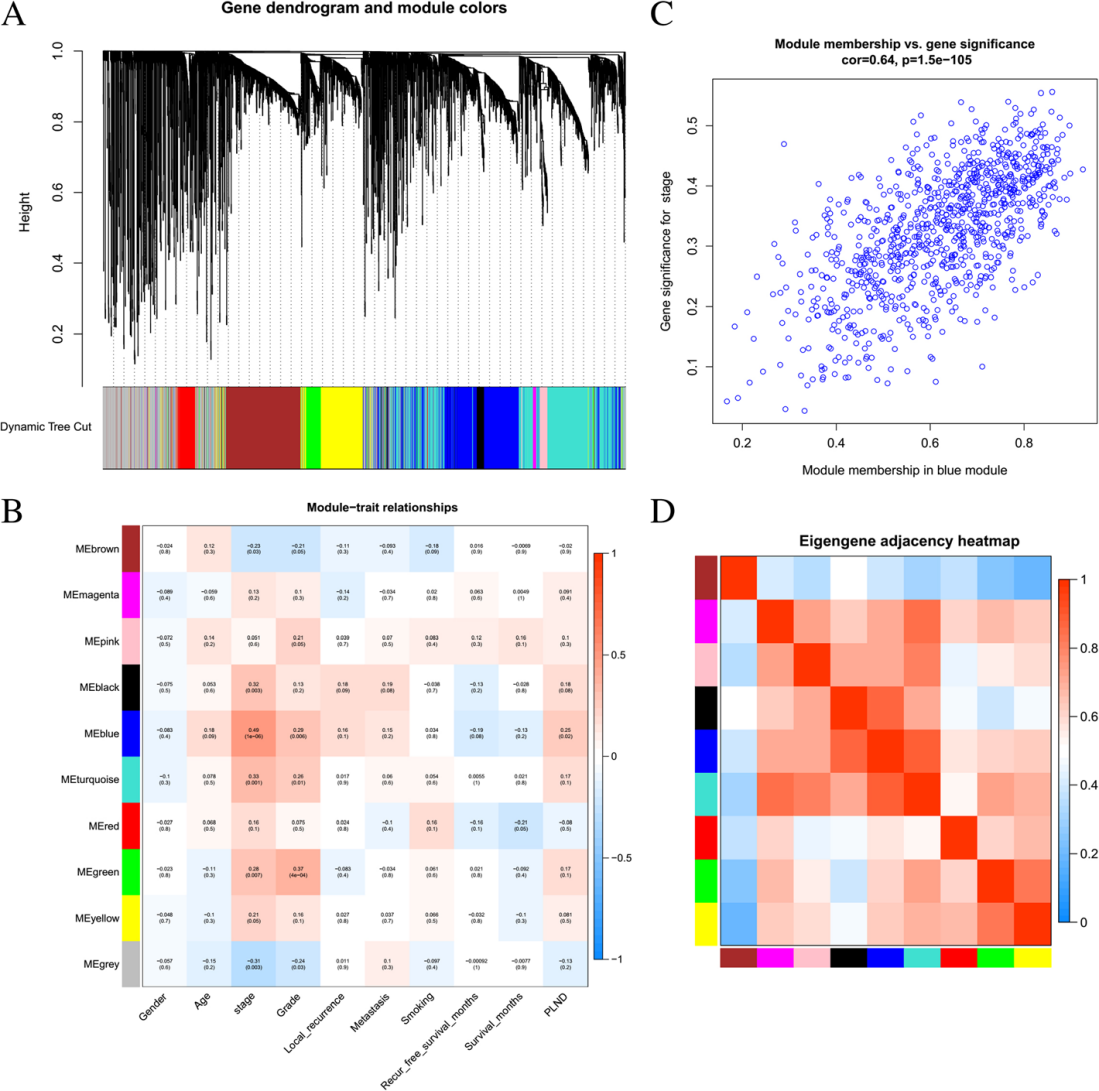
The genes enrichment and module identification. **a** Dendrogram of all filtered genes enriched according to a dissimilarity measure (1-TOM) and the cluster module colors. **b** Heatmap of the correlation between the clinical traits and MEs of bladder cancer. The darker the module color, the more significant their relationship. **c** The scatter plot between the blue module membership and the gene significance for tumor staging. **d** Eigengene adjacency heatmap

The sample dendrogram and trait heatmap are shown in Fig. 1a. As mentioned above, the samples of GSM786521, GSM786580, GSM786492, and GSM786537 were excluded. The R package of “WGCNA” was used to classify 13,222 genes with similar expression levels into different modules. In our research, *β* = 8 was set to guarantee high scale independence (near 0.9) and low mean connectivity (near 0). The dissimilarity of the modules was set as 0.2, and a total of 10 modules were generated (Fig. 2a). The module trait relationship is shown in Fig. 2b. The blue module associated with the tumor staging was the deepest(*cor* = 0.49, *P* = 1E-06), which was chosen for further analysis(Fig. 2c). The module membership in the blue module and the gene significance have a high correlation(0.64) and high *P*-value(1.5E-105), suggesting that the module is suitable for identifying the hub genes associated with the staging of cancer. The eigengene adjacency heatmap is shown in Fig. 2d, which indicates that the blue module, the black module, and some other modules were adjacent.

### Co-expression network construction and hub genes identification

**Fig. 3:**
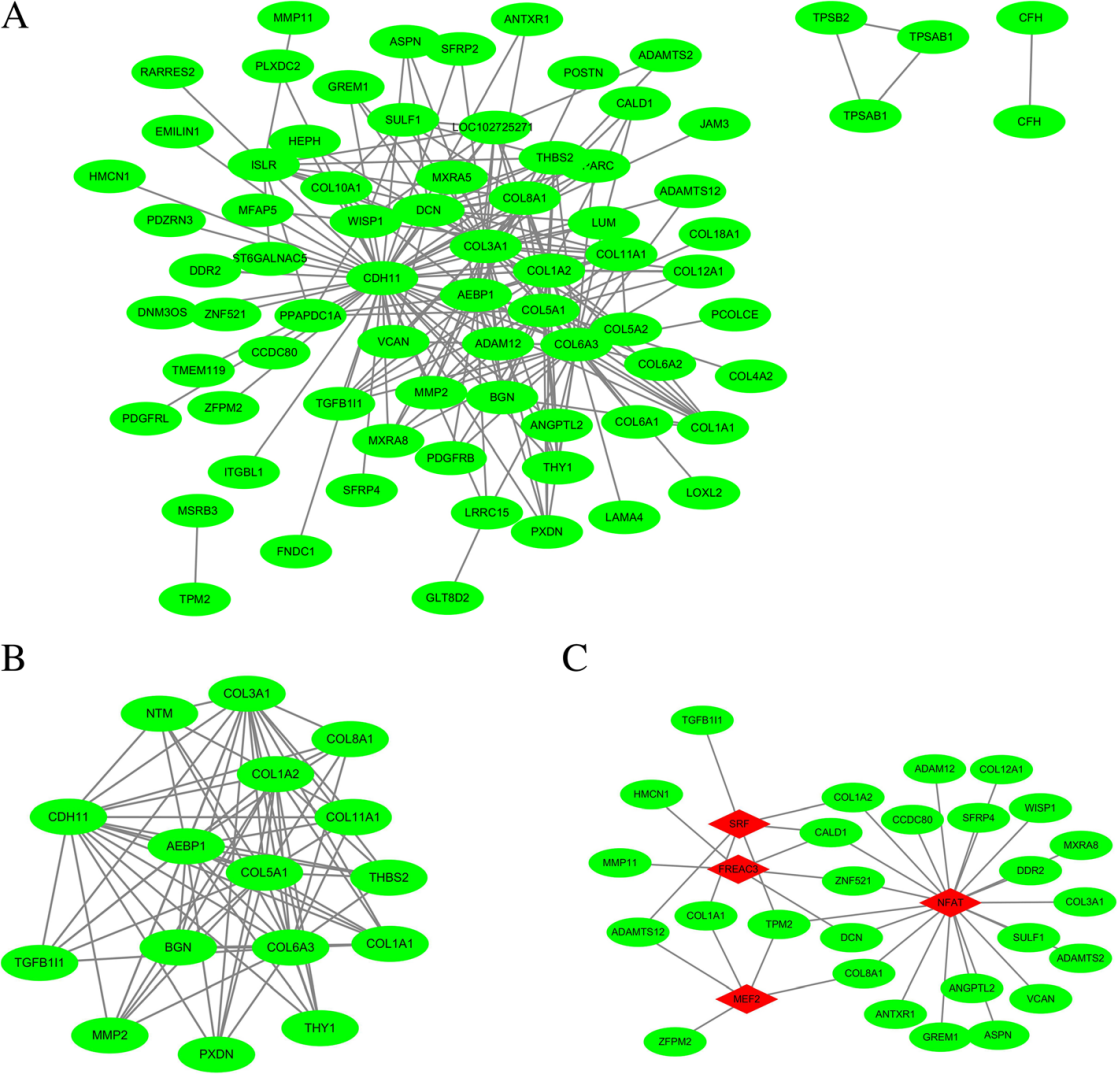
The co-expression network, hub cluster and its associated TFs. **a** The co-expression network of the significant genes in the blue module. It has 76 nodes and 208 edges. **b** The most significant cluster generated by MCODE. It has 16 nodes and 77 edges. **c** The transcriptional factor (*TF*) network of the 76 genes generated by Webgestalt. The diamond and red color indicate the TFs while the green and eclipse shape indicate the target protein

The edges signifying the correlations in the blue module were filtered by a condition of the weight value being greater than 0.2, and a total of 208 edges were obtained. Seventy-six nodes were identified after inputting them into Cytoscape (Fig. 3a). MCODE was applied to discover the hub clusters in the network. The most significant cluster was shown in Fig. 3b. Its degree, neighborhood connectivity, and other parameters were shown in Table 2. The identified hub genes were *CDH11, COL3A1, COL6A3, COL5A1, AEBP1, COL1A2, NTM, COL11A1, THBS2, COL8A1, COL1A1, BGN, MMP2, PXDN, THY1,* and *TGFB1I1*. In addition, we input the 76 genes into the WebGestalt with the *P*-value< 0.01 and discovered four transcriptional factors including *NFAT, SRF, FREAC3,* and *MEF2*. Its network with the proteins was presented in Fig. 3c.

**Table 2:** The16 hub genes identified by MCODE (ranked by degree)

Gene Name	Degree	Neighborhood Connectivity	Average Shortest Path Length	Closeness Centrality	Clustering Coefficient	Stress	Topological Coefficient
*CDH11*	49	6.244898	1.27941176	0.7816092	0.089286	4554	0.115646
*COL3A1*	35	8.971429	1.5	0.66666667	0.191597	2022	0.133902
*COL6A3*	31	9.16129	1.55882353	0.64150943	0.197849	1944	0.141633
*COL5A1*	25	10.72	1.66176471	0.60176991	0.266667	1420	0.16625
*AEBP1*	21	12.61905	1.70588235	0.5862069	0.347619	852	0.190476
*COL1A2*	19	14	1.75	0.57142857	0.432749	464	0.212121
*NTM*	11	19.81818	1.85294118	0.53968254	0.418182	544	0.295794
*COL11A1*	10	20.7	1.86764706	0.53543307	0.511111	388	0.312121
*THBS2*	10	21.6	1.86764706	0.53543307	0.488889	432	0.322388
*COL8A1*	10	21.6	1.86764706	0.53543307	0.488889	432	0.322388
*COL1A1*	9	21.55556	1.91176471	0.52307692	0.694444	194	0.331624
*BGN*	8	24.5	1.92647059	0.51908397	0.964286	2	0.376923
*MMP2*	7	26.85714	1.94117647	0.51515152	1	0	0.413187
*PXDN*	6	30	1.95588235	0.5112782	1	0	0.461538
*THY1*	6	30	1.95588235	0.5112782	1	0	0.461538
*TGFB1I1*	6	30	1.95588235	0.5112782	1	0	0.461538

### Function annotation of the module of interest and the hub genes

**Fig. 4:**
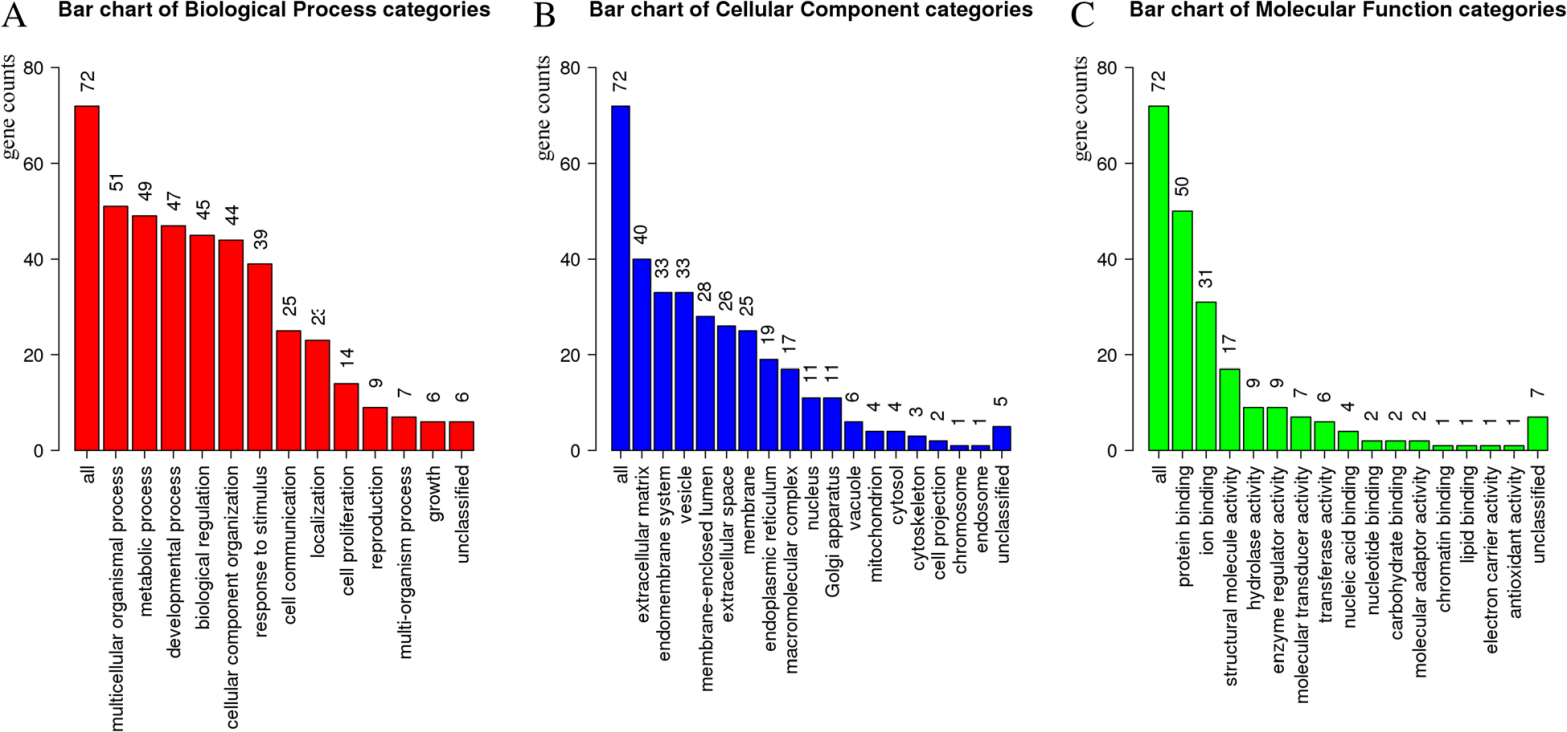
The gene ontology enrichment of the significant genes in the blue module. It contains three categories including biological process (**a**), cellular component (**b**) and molecular function (**c**). The vertical axis indicates the number of enriched genes

The results of the GO analysis of the 76 genes in WebGestalt were shown in Fig. 4. They were classified into three groups including the biological process, the molecular function, and the cellular component. It was found that the genes were associated with multicellular organism process, extracellular matrix, protein binding and so forth. Table 3 demonstrated the results regarding the hub genes enriched by BiNGO. These genes were suggested related to collagen fibril organization, blood vessel development, cell adhesion, extracellular matrix organization, and collagen biosynthetic process.

**Table 3:** The top 15 GO items of the 16 hub genes provided by BINGO

GO-ID	P-value	Description	Genes in the test set
30,199	7.08-E-11	collagen fibril organization	*COL1A1,COL3A1,COL1A2,COL5A1,COL11A1*
1568	7.94E-11	blood vessel development	*COL1A1,COL3A1,COL1A2,COL5A1,MMP2,BGN,COL8A1,THY1*
7155	2.19E-10	cell adhesion	*COL3A1,COL5A1,COL11A1,TGFB1I1,CDH11,COL6A3,COL8A1,AEBP1,THY1,THBS2*
30,198	6.59E-10	extracellular matrix organization	*COL1A1,COL3A1,COL1A2,COL5A1,COL11A1,PXDN*
32,964	1.94E-08	collagen biosynthetic process	*COL1A1,COL3A1,COL5A1*
32,963	2.59E-08	collagen metabolic process	*COL1A1,COL3A1,COL5A1,MMP2*
43,588	4.26E-08	skin development	*COL1A1,COL3A1,COL1A2,COL5A1*
44,236	7.36E-08	multicellular organismal metabolic process	*COL1A1,COL3A1,COL5A1,MMP2*
48,513	9.99E-08	organ development	*COL1A1,COL3A1,COL1A2,COL5A1,MMP2,TGFB1I1,BGN,COL6A3,COL8A1,AEBP1,THY1*
48,731	1.52E-07	system development	*COL1A1,COL3A1,COL1A2,COL5A1,MMP2,TGFB1I1,CDH11,BGN,COL6A3,COL8A1,AEBP1,THY1*
1501	6.53E-07	skeletal system development	*COL1A1,COL3A1,COL1A2,MMP2,CDH11,AEBP1*
60,346	2.05E-06	bone trabecula formation	*COL1A1,MMP2*
9653	1.03E-05	anatomical structure morphogenesis	*COL1A1,COL1A2,COL5A1,MMP2,TGFB1I1,BGN,COL8A1,THY1*
32,501	1.11E-05	multicellular organismal process	*COL11A1,MMP2,TGFB1I1,BGN,AEBP1,THY1,COL1A1,COL3A1,COL1A2,COL5A1,CDH11,COL6A3,COL8A1*
43,589	1.28E-05	skin morphogenesis	*COL1A1,COL1A2*

Ranked by *P-value*

### Survival analysis and significant gene identification

**Fig. 5:**
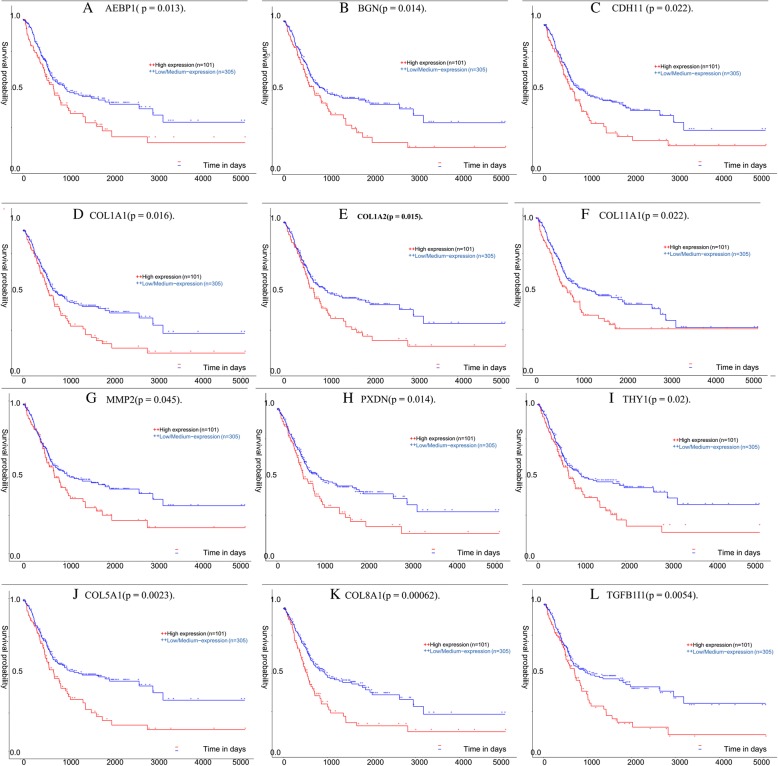
Survival plot of the significant genes by Kaplan Meier test. The data was extracted from the TCGA website with n signifying the number of patients and BLCA signifying bladder carcinoma. The Kaplan Meier test *P*-value< 0.05: **a** *AEBP1.* **b** *BGN.* **c** *CDH11,* (**d**) *COL1A1,* (**e**) *COL1A2,* (**f**) *COL11A1,* (**g**) *MMP2,* (**h**) *PXDN,* and (**i**) *THY1*. The test P-value< 0.01: **j** *COL5A1,* (**k**) *COL8A1 and* (**l**) *TGFB1I1*

**Fig. 6:**
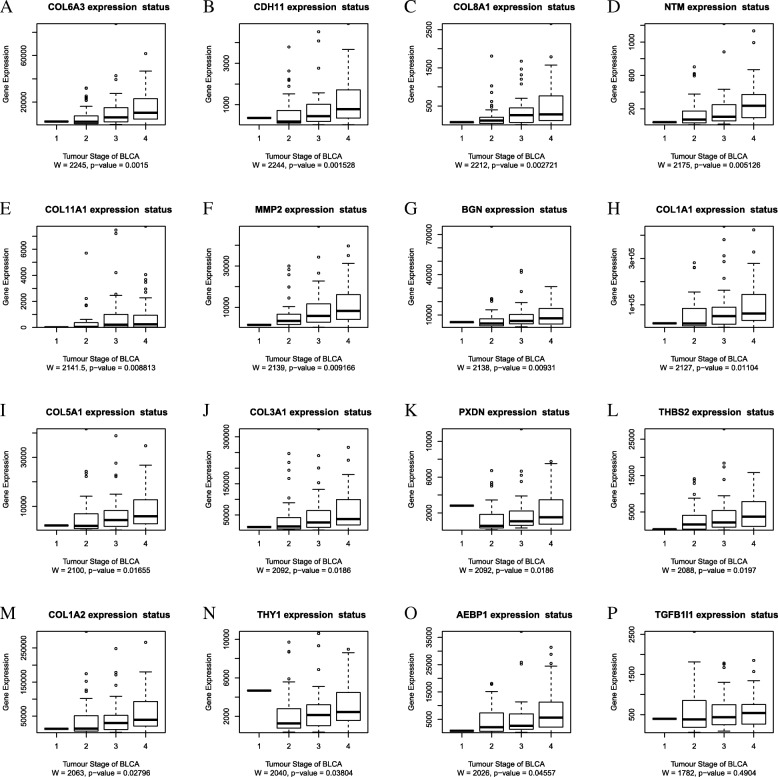
The mRNA expression of 16 hub genes in different tumor stage of TCGA patients. The stage III and stage IV were set high degree stage group and the stage I and stage II were set low degree stage group. The Wilcoxon test was done between the two groups. The genes whose *P*-value ranked from low to high were as follows: **a** *COL6A3.* **b** *CDH11.* **c** *COL8A1.* **d** *NTM.* **e** *COL11A1.* **f** *MMP2.* **g** *BGN.* **h** *COL1A1.* **i** *COL5A1.* **j** *COL3A1.* **k** *PXDN.* **l** *THBS2.* **m** *COL1A2.* **n** *THY1.* **o** *AEBP1.* **p** *TGFB1I1*. Except for the expression of *TGFB1I1*, others were significant in distinguishing low and high tumor stage (*P*-value< 0.05)

The bladder cancer expression data in TCGA was analyzed by the UALCAN (http://ualcan.path.uab.edu/), and the Kaplan-Meier curves for the overall survival of TCGA patients with bladder cancer were obtained according to the low and high expression of the 16 hub genes. The genes with *P*-values less than 0.01 were *COL5A1, COL8A1,* and *TGFB1I1*(Fig. 5) while those with P-values between 0.05 and 0.01 were *AEBP1, CDH11, COL1A1, COL1A2, COL11A1, MMP2, PXDN, BGN,* and *THY1*(Fig. 5). 75% of the genes in the cluster suitable for prognosis prediction of the patients with bladder cancer (*P* < 0.05), suggested that the cluster was important for bladder cancer. The high expression of *COL5A1, COL8A1, BGN, TGFB1I1, AEBP1, CDH11, COL1A1, COL1A2, COL11A1, MMP2, PXDN,* and *THY1* was related to the poor prognosis of the disease. As such, *COL5A1, COL8A1* and *TGFB1I1* can serve as the prognosis biomarkers for patients of bladder cancer. The boxplots demonstrating the correlations between the tumor staging and the hub genes were shown in Fig. 6. The significance of the gene expression in low and high stages was examined by the Wilcoxon test. Except for *TGFB1I1*, others were significant in distinguishing low and high stage of bladder cancers(P < 0.05).

## Discussion

Bladder cancer was one of the most common cancers worldwide [1]. Lower-stage tumors were smaller and had a better chance of successful treatment whereas patients with higher-stage cancers may suffer a poor prognosis [29]. With the advancement of science and biological technology, the accuracy treatment of bladder cancer was gaining momentum, and its development called for the identification of the hub genes relating to bladder cancer. WGCNA was often used to relate modules to external clinical traits and identified the important genes of a tumor. Deng, S. P. et al. [16] had identified *“GDF9”, “CYP1A2”, “ATF7”, “TRPM3”, “CER1”, “PTPRJ”, “KCNIP1”, and “LRRC15”* as hub genes in bladder cancer by construction and estimation of two DCNs(normal and cancer state) in 2015. Gaballah [17] identified candidate genes: PURA, SRPK2, TRAK1, BRD2, and UPF3 in progression and invasiveness of bladder carcinoma based on DEGs acquired by comparing invasive and noninvasive samples by Limma R packages in 2016. Zhang, X. et al. [30] revealed *POU2F3*, *NKD1*, *CYP2C8, LINC00189*, *GCC2*and *OR9Q1* were several remarkable “hub genes” in squamous cell carcinoma of urinary bladder in 2016. The samples were single cells from their own hospital, the DEGs were acquired by NOISeq R packages, and the WGCNA of bladder cancer were focused on intra-tumor heterogeneity measured by coefficient of variation (CV). Yuan, Lushun et al. [31] have reported that the overexpression of COL3A1 confers a poor prognosis in human bladder cancer in 2017.The DEGs used for WGCNA were obtained by comparing low stage bladder cancers (Ta-T1) and high stage bladder cancers (T2-T4) by Limma R packages, and hub genes were defined by module connectivity. Li, S. et al. [15] reported *MMP11, COL5A2, CDC25B, TOP2A, CENPF, CDCA3, TK1, TPX2, CDCA8, AEBP1, and FOXM1* correlated with clinical prognosis of patients with bladder cancer in 2017. The gene chip GSE13507 they used contained 23 recurrent non-muscle invasive tumor tissues, 58 normal looking bladder mucosae surrounding cancer and 10 normal bladder. Probesets were filtered by their variance across all samples, and hub genes were identified using a networkScreening function. Giulietti, M et al. [18] have reviewed the literature of WGCNA analysis in bladder cancer and concluded hub miRNAs of miR-1-1/2, miR-28, miR-133a-1/2, miR-139, miR-143, miR-195, and miR-6507 in the disease by WGCNA in 2018. However, they choose to perform WGCNA only on DEG genes which may easily obtain the positive results and increase the possibility of leaving out the lowly expressed but highly correlated genes. In the current study, the gene chip GSE31674 including its clinical traits was downloaded from the GEO datasets. In order to avoid the disadvantages and make our results more accurate, all the filtered 13,222 genes were used for WGCNA analysis. We finally obtained 16 hub genes associated the staging of bladder cancer, including *CDH11, COL3A1, COL6A3, COL5A1, AEBP1, COL1A2, NTM, COL11A1, THBS2, COL8A1, COL1A1, BGN, MMP2, PXDN, THY1, and TGFB1I1*. These genes were associated with the process of collagen fibril organization(GO:0030199)(COL1A1,COL3A1,COL1A2,COL5A1,COL11A1),blood vessel development(GO:0001568)(COL1A1,COL3A1,COL1A2,COL5A1,MMP2,BGN,COL8A1,THY1),cell adhesion(GO:0007155)(COL3A1,COL5A1,COL11A1,TGFB1I1,CDH11,COL6A3,COL8A1,AEBP1,THY1,THBS2), extracellular matrix organization(GO:0030198)(COL1A1,COL3A1,COL1A2,COL5A1,COL11A1,PXDN), collagen biosynthetic process(GO:0032964)(COL1A1,COL3A1,COL5A1) in bladder cancer. They were also associated with the pathways of ECM-receptor interaction(hsa04512)(COL3A1, COL6A3, COL1A2, COL1A1, COL11A1, THBS2, COL5A1), protein digestion and absorption(hsa04974)(COL3A1, COL6A3, COL1A2, COL1A1, COL11A1, COL5A1),focal adhesion(hsa04510)(COL3A1, COL6A3, COL1A2, COL1A1, COL11A1, THBS2, COL5A1),and PI3K-Akt signaling pathway(hsa04151)(COL3A1, COL6A3, COL1A2, COL1A1, COL11A1, THBS2, COL5A1) in bladder cancer. The expression levels of most of these genes had positive correlations with tumor staging. After examination by the TCGA datasets, *THY1, AEBP1, CDH11, COL1A1, COL1A2, COL11A1, MMP2, PXDN, BGN, COL5A1, COL8A1, and TGFB1I1* were associated with the patient’s prognosis(*P* < 0.05). The *P*-values of *COL5A1, COL8A1,* and *TGF B1I1* were less than 0.01 and were then thought important among them. However, *TGFB1I1* was meaningless in revealing tumor stage and was excluded. *COL5A1* [32–35] encoded an alpha chain which was closely related to type XI collagen and was reported contributing to the metastasis of lung adenocarcinoma. *COL8A1* [36–38] was a short α-chains of type VIII collagen, which was connected to angiogenesis and vascular remodeling; it was reported playing an important role in hepatocarcinoma cells. In our study, we find it was also associated with bladder cancer.

## Conclusion

After the weighted gene co-expression network analysis of bladder cancer and the examination of the hub genes, we find *THY1, AEBP1, CDH11, COL1A1, COL1A2, COL11A1, MMP2, PXDN, BGN, COL5A1, COL8A1* associated with the tumor stage as well as tumor patients’ prognosis. *COL5A1, COL8A1* were most significant in the prediction of the bladder cancer’s prognosis(*P*-value< 0.01), and may serve as the therapeutic target for the disease.

## Abbreviations

BGN: matrix-remodelling associated 8
BLCA: bladder carcinoma
BP: biological process
CC: cell component
COL5A1: collagen type V alpha 1 chain
*COL8A1*: collagen type *VIII* alpha 1 chain
DCNs: differential co-expression networks
GS: gene significance
MF: molecular function
MM: module membership
TGFB1I1: transforming growth factor beta 1 induced transcript 1
TOM: topological overlap measure
WGCNA: weighted correlation network analysis
